# The Value of Applying Machine Learning in Predicting the Time of Symptom Onset in Stroke Patients: Systematic Review and Meta-Analysis

**DOI:** 10.2196/44895

**Published:** 2023-10-12

**Authors:** Jing Feng, Qizhi Zhang, Feng Wu, Jinxiang Peng, Ziwei Li, Zhuang Chen

**Affiliations:** 1 Department of Neurology Fifth People’s Hospital of Jinan Jinan China; 2 Department of Pulmonary Disease and Diabetes Mellitus Central Hospital of Enshi Tujia and Miao Autonomous Prefecture Enshi China; 3 Medical Department Hubei Enshi College Enshi China; 4 Experimental Center Shandong University of Traditional Chinese Medicine Jinan China; 5 Department of Cardiovascular Medicine Fifth People’s Hospital of Jinan Jinan China

**Keywords:** machine learning, ischemic stroke, onset time, stroke

## Abstract

**Background:**

Machine learning is a potentially effective method for identifying and predicting the time of the onset of stroke. However, the value of applying machine learning in this field remains controversial and debatable.

**Objective:**

We aimed to assess the value of applying machine learning in predicting the time of stroke onset.

**Methods:**

PubMed, Web of Science, Embase, and Cochrane were comprehensively searched. The C index and sensitivity with 95% CI were used as effect sizes. The risk of bias was evaluated using PROBAST (Prediction Model Risk of Bias Assessment Tool), and meta-analysis was conducted using R (version 4.2.0; R Core Team).

**Results:**

Thirteen eligible studies were included in the meta-analysis involving 55 machine learning models with 41 models in the training set and 14 in the validation set. The overall C index was 0.800 (95% CI 0.773-0.826) in the training set and 0.781 (95% CI 0.709-0.852) in the validation set. The sensitivity and specificity were 0.76 (95% CI 0.73-0.80) and 0.79 (95% CI 0.74-0.82) in the training set and 0.81 (95% CI 0.68-0.90) and 0.83 (95% CI 0.73-0.89) in the validation set, respectively. Subgroup analysis revealed that the accuracy of machine learning in predicting the time of stroke onset within 4.5 hours was optimal (training: 0.80, 95% CI 0.77-0.83; validation: 0.79, 95% CI 0.71-0.86).

**Conclusions:**

Machine learning has ideal performance in identifying the time of stroke onset. More reasonable image segmentation and texture extraction methods in radiomics should be used to promote the value of applying machine learning in diverse ethnic backgrounds.

**Trial Registration:**

PROSPERO CRD42022358898; https://www.crd.york.ac.uk/Prospero/display_record.php?RecordID=358898

## Introduction

Stroke is the second leading cause of disability and death; it makes the largest contribution to global neurological disability-adjusted life-years, with an estimated prevalence of 80.1 million cases in 2016 [1]. Current treatments for stroke are mainly thrombolytic therapy and intravascular thrombectomy [2-4]. The effect of thrombolytics varies with the time of treatment. Thrombolytic treatment within a specific time window can be effective, but the treatment is ineffective if it is outside the time window. Stroke treatment is urgent, with a recommended time window within 4.5 or 6 hours [5,6]. Unfortunately, patients with an unclear time of stroke onset are common in clinical practice [7]. Approximately a quarter of patients with acute stroke are not sure when their symptoms began, which causes enormous obstacles in selecting clinical medical strategies [8,9].

Nowadays, the identification of the time of stroke onset mainly depends on magnetic resonance imaging (MRI). By screening brain tissue comprehensively, MRI can detect ischemia-induced hydrodynamic changes and ischemic tissue, which is helpful for clinicians to assess the pathological features of oversize, swelling, and bleeding [10]. MRI-based techniques have been adopted to stratify stroke patients with an unknown time of onset. However, image evaluation generally relies on clinical experience, which is inevitably subjective [11]. Existing evidence has shown that the performance of imaging in estimating stroke onset time needs to be improved [12]. Heterogeneity in image resolution and medical workers’ experience compromises the reliability of imaging in predicting the time of stroke onset [13,14]. Therefore, more objective approaches are needed to accurately identify the time of symptom onset in stroke patients.

Machine learning has been introduced for the prediction of the onset time of acute ischemic stroke in recent years [15-18]. Numerous studies on the application of machine learning in identifying and predicting the time of stroke onset have been published [19-21].

However, the heterogeneity between different studies cannot be ignored, given the diversity of machine learning methods and the differences in included modeling indicators. Whether machine learning can effectively predict stroke onset time remains hotly debated. Therefore, this systematic review and meta-analysis aimed to explore the value of applying machine learning in predicting the time of symptom onset in stroke patients.

## Methods

This systematic review and meta-analysis was reported in accordance with the PRISMA (Preferred Reporting Items for Systematic reviews and Meta-Analyses) guidelines. The protocol for this study was registered on PROSPERO (CRD42022358898).

### Literature Search

We searched the PubMed, Web of Science, Embase, and Cochrane databases comprehensively from their inception to July 24, 2022. A combination of keywords and subject headings was used for the search, and the language was restricted to English. The main search terms included *stroke*, *cerebrovascular accident*, *apoplexy*, *machine learning*, *onset*, and *stroke time*. The specific search strategy is presented in Multimedia Appendix 1. Two researchers independently searched the literature, and any disagreements were solved by a third researcher. We performed a further, manual search of the reference lists of the included articles to find potential eligible studies.

### Eligibility Criteria

After removing duplicates using EndNote (Thomson ResearchSoft), we screened the literature according to the inclusion and exclusion criteria presented in Textbox 1.

**Inclusion and exclusion criteria.**

**Inclusion criteria**

- Population: ischemic stroke patients
- Outcomes: (1) the machine learning prediction model for stroke onset time was completely constructed; (2) research was included on different machine learning models in the same data set
- Research types: case-control studies, cohort studies, nested case-control studies, and case-cohort studies
- Language: English
- Other criteria: Studies without external validation

**Exclusion criteria**

- Outcomes: (1) Only the influencing factors were analyzed and a complete risk model was not built; (2) the following outcome indicators were missing: receiver operating characteristics curve, C statistic, C index, sensitivity, specificity, accuracy, recovery, confusion matrix, diagnostic quad table, F_1_-score, calibration curve
- Research types: meta-analyses, reviews, guides, and expert opinions
- Language: other than English
- Other criteria: (1) studies with few samples (<50 cases), (2) validation studies of maturity scales, (3) studies on the accuracy of single-factor prediction

### Literature Screening and Data Extraction

The retrieved documents were imported into EndNote X9 for management. The titles and abstracts were screened to exclude irrelevant studies. The full texts of the remaining articles were downloaded and read to select eligible studies. Before data extraction, we prepared a standard electronic information extraction form. The primary outcomes included in the study are C index, sensitivity, and specificity. The abovementioned literature screening and information extraction were independently carried out by 2 researchers, and any disagreement was resolved by a third researcher.

### Risk of Bias Assessment

The PROBAST (Prediction Model Risk of Bias Assessment Tool) was used [22] to evaluate the risk of bias in the included studies. This tool contains several questions across 4 different domains: participants, predictors, outcome, and analysis. The overall applicability of the included studies was also assessed.

The 4 domains comprise 2, 3, 6, and 9 specific questions, respectively, and each question was answered as “yes,” “probably yes,” “no,” “probably no,” or “no information.” If a domain included at least 1 response of “no” or “probably no,” it was considered at high risk. A domain with all the responses being “yes” or “probably yes” was judged low risk. When all domains were considered low risk, the overall risk of bias was rated low. When at least 1 domain was considered high risk, the overall risk of bias was high. Two researchers independently carried out the risk of bias assessment using PROBAST and cross-checked their results. If there was any dispute, a third researcher was consulted to assist in the final decision.

### Statistical Analysis

We conducted a meta-analysis of the evaluation indicators (C index and accuracy) of the machine learning models. If the C index had no 95% CI or SE, we estimated the SE with the calculation method proposed by Debray et al [23]. Given different variables and inconsistent parameters in learning models, a random effects model was preferred in the meta-analysis of the C index. In addition, we used a bivariate mixed effects model to perform the meta-analysis of sensitivity and specificity. The meta-analysis of our study was conducted using R (version 4.2.0, R Development Core Team).

## Results

### Study Selection

**Figure 1:**
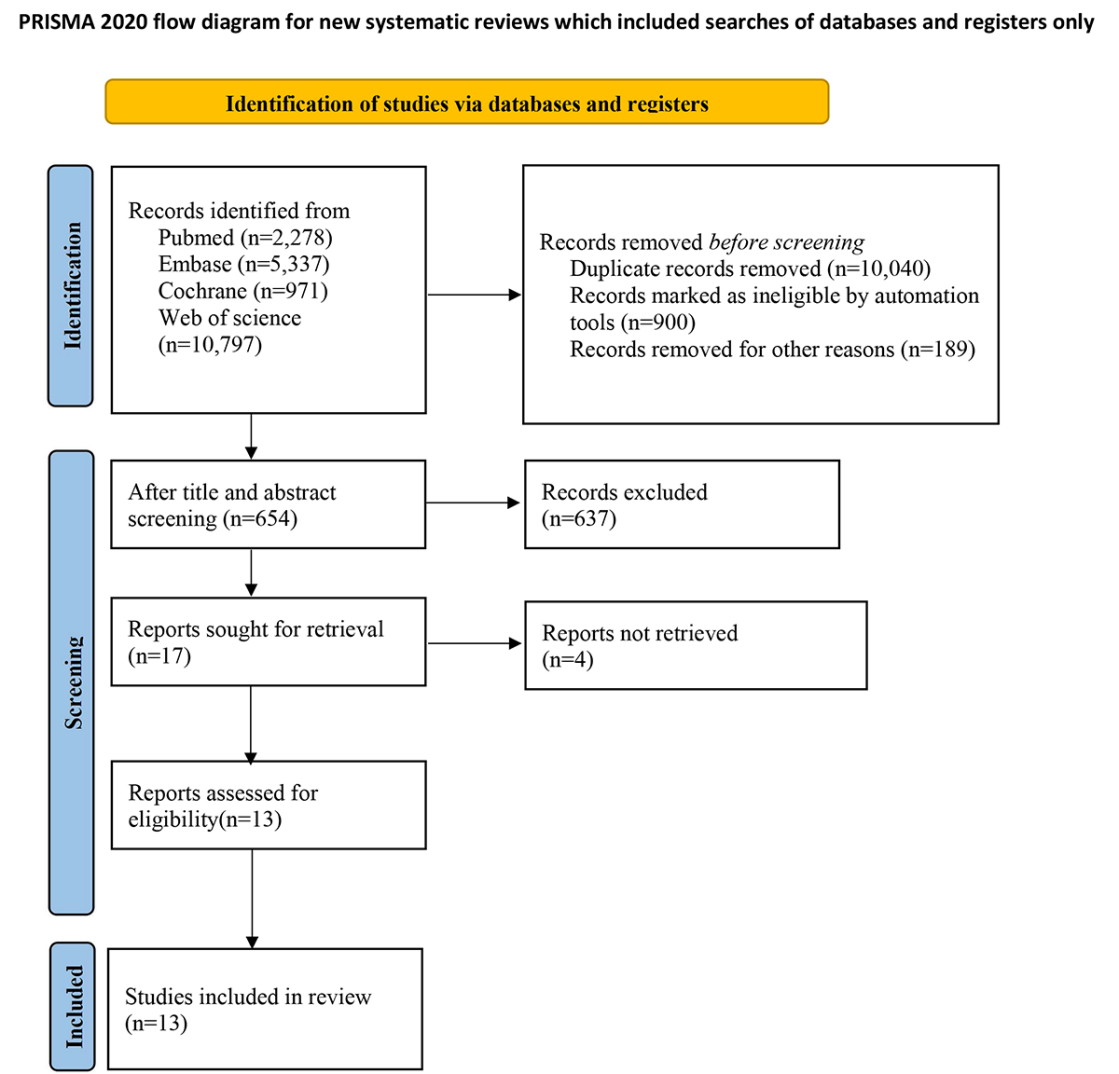
Flow chart of study selection process for studies included in and excluded from the meta-analysis.

After removing the duplicates, a total of 843 papers were left. Based on the title and abstract screening, the full texts of 17 papers were downloaded and read. Finally, 13 eligible papers were included in this study. The details of the literature screening process are shown in Figure 1.

### Study Characteristics

There were 11 case-control studies and 2 cohort studies. Among the included studies [17,18,24-34], 5 were from China, 3 from Belgium, 4 from the United States, and 1 from South Korea (shown in Table 1) There were 55 models involved, with 41 models in the training set and 14 in the validation set. These models were mostly based on logistic regression (LR), with radiomics-based modeling variables.

**Table 1:** Characteristics of included studies for the value of applying machine learning in predicting the time of symptom onset in stroke patients.

Study, year	Country	Study type	Patient source	N (total)	Onset time (hours)	N1^a^, participants, n	N2^b^, participants, n	Validation set generation method	N3^c^, participants, n	Modeling variables	Type of model
Wouters et al [24], 2016	Belgium	Case-control	Multicenter	141	<4.5,<3,<6	50,15,92	141	Leave-one-out	N/A^d^	MRI^e^	LR^f^
Zhu et al [25], 2021	China	Case-control	Multicenter	268	≤4.5,>4.5	173,95	N/A	Random sampling plus external verification	N/A	MRI	SVM^g^, LR, RF^h^, GBDT^i^, DT^j^
Zhang et al [17], 2022	China	Case-control	Single-center	84	≤4.5,>4.5	58,26	51	Random sampling	33	MRI	DL^k^, XGBoost^l^, GBM^m^, GLM^n^, RF, DT
Zhang et al [26], 2021	US	Case-control	Single-center	422	N/A	N/A	340	Random sampling	82	MRI	CNN^o^
Yao et al [27], 2020	China	Case-control	Single-center	316	≤4.5,>4.5	N/A	156,81	Random sampling	52,27	CT^p^	LR
Wouters et al [28], 2017	Belgium	Cohort study	Register database	181	≤6 ,＞6	124,57	69,22	External verification	55,35	MRI	LR
Wouters et al [29], 2018	Belgium	Cohort study	Single-center	318	4.5	N/A	N/A	N/A	N/A	MRI	LR
Wen et al [30], 2021	China	Case-control	Multicenter	123	≤4.5,>4.5	46,77	85	Random sampling plus external verification	38,60	CT	LR
Polson et al [31], 2021	US	Case-control	N/A	772	N/A	N/A	N/A	External verification	N/A	MRI	DT
Lee et al [18], 2020	Korea	Case-control	Single-center	355	4.5	355	299	Random sampling	56	MRI	LR, RF, SVM
Jiang et al [32], 2022	China	Case-control	Multicenter	554	4.5	N/A	N/A	N/A	N/A	MRI	SVM, NB^q^, KNN^r^, AdaBoost, ANN^s^
Ho et al [33], 2019	US	Case-control	Single-center	131	≤4.5,>4.5	85,46	N/A	Cross-validation	N/A	MRI	LR, RF, XGBoost, SVM
Ho et al [34], 2017	US	Case-control	Register database	105	<4.5,≥4.5	83,22	N/A	N/A	N/A	MRI	SVM, RF, XGBoost

^a^N1: people trained at the time of stroke onset. Values separated by commas refer to the number people with stroke onset at the respective times in the “onset time” column.

^b^N2: training set. Values separated by commas refer to the number people with stroke onset at the respective times in the “onset time” column.

^c^N3: validation set. Values separated by commas refer to the number people with stroke onset at the respective times in the “onset time” column.

^d^N/A: not applicable.

^e^MRI: magnetic resonance imaging.

^f^LR: logistic regression.

^g^SVM: support vector machine.

^h^RF: random forest.

^i^GBDT: gradient-boosted decision tree.

^j^DT: decision tree.

^k^DL: deep learning.

^l^XGBoost: extreme gradient boosting.

^m^GBM: gradient boosting machine.

^n^GLM: generalized linear model.

^o^CNN: convolutional neural network.

^p^CT: computed tomography.

^q^NB: naive Bayes.

^r^KNN: k-nearest neighbor.

^s^ANN: artificial neural network.

### Quality of Evidence and Risk of Bias

**Figure 2:**
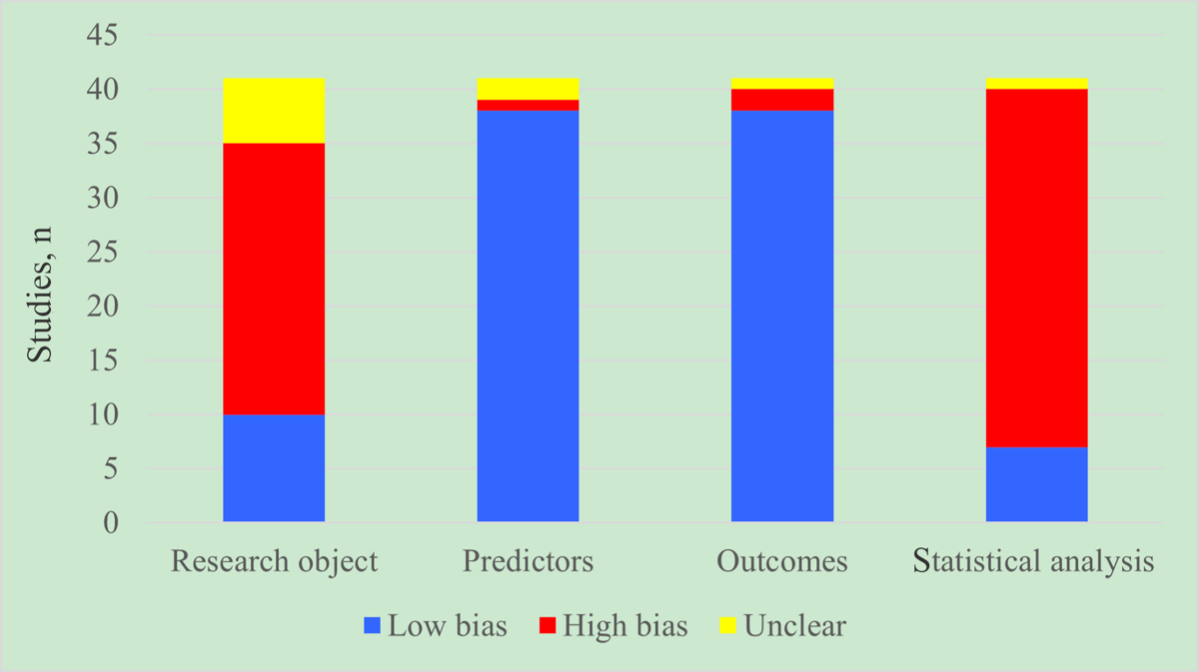
Risk of bias assessment for studies included in the meta-analysis.

All 41 models in the training set were radiomics-based machine learning models. The inclusion and exclusion criteria for the cases were relatively reasonable. In addition, the onset time records were suitably reliable. In terms of data generation, only one study described the processing method for missing clinical values. Of note, no missing values in the radiomics data were mentioned. In the modeling process, the verification set was mostly generated through random sampling for internal verification, while 3 multicenter studies had independent external verification sets. There were fewer than 100 samples in the verification set. The risk of bias assessment of the included studies is presented in Figure 2.

### Statistical Analysis

#### C Index

There were a total of 3770 participants and 55 machine learning models, with 41 models in the training set and 14 in the validation set. The meta-analysis of the C index in the training set and in the validation set included 13 studies and 6 studies, respectively. The overall C index was 0.800 (95% CI 0.773-0.826) in the training set and 0.781 (95% CI 0.709-0.852) in the validation set. The meta-analysis of the C index is shown in Table 2.

**Table 2:** The C index results for the value of applying machine learning in predicting the time of symptom onset in stroke patients.

Subgroup			Training set					Testing set		
			Studies, n		C index (95% CI)		Studies, n			C index (95% CI)
**Model**										
	Logistic regression	12		0.807 (0.741-0.872)		7			0.807 (0.691-0.923)	
	Support vector machine	8		0.782 (0.724-0.839)		1			0.895 (0.854-0.936)	
	Artificial neural network	2		0.798 (0.689-0.907)		N/A^a^			N/A	
	Boosting	6		0.787 (0.697-0.877)		2			0.714 (0.580-0.849)	
	Deep learning	1		0.900 (0.816-0.984)		1			0.754 (0.584-0.924)	
	Random forest	6		0.795 (0.733-0.856)		1			0.697 (0.502-0.893)	
	Decision tree	3		0.822 (0.721-0.923)		1			0.681 (0.488-0.874)	
	Generalized linear model	1		0.798 (0.687-0.909)		1			0.662 (0.459-0.865)	
	K-nearest neighbor	1		0.797 (0.762-0.832)		N/A			N/A	
	Naive Bayes	1		0.802 (0.767-0.837)		N/A			N/A	
**Onset time (hours)**										
	≤4.5 hours	38		0.80 (0.77-0.83)		13			0.79 (0.71-0.86)	
	≤6 hours	2		0.76 (0.71-0.81)		1			0.73 (0.62-0.84)	
	≤3 hours	1		0.90 (0.81-0.99)		N/A			N/A	
Overall			41		0.800 (0.773-0.826)		14			0.781 (0.709-0.852)

^a^N/A: not applicable.

#### Sensitivity and Specificity

**Figure 3:**
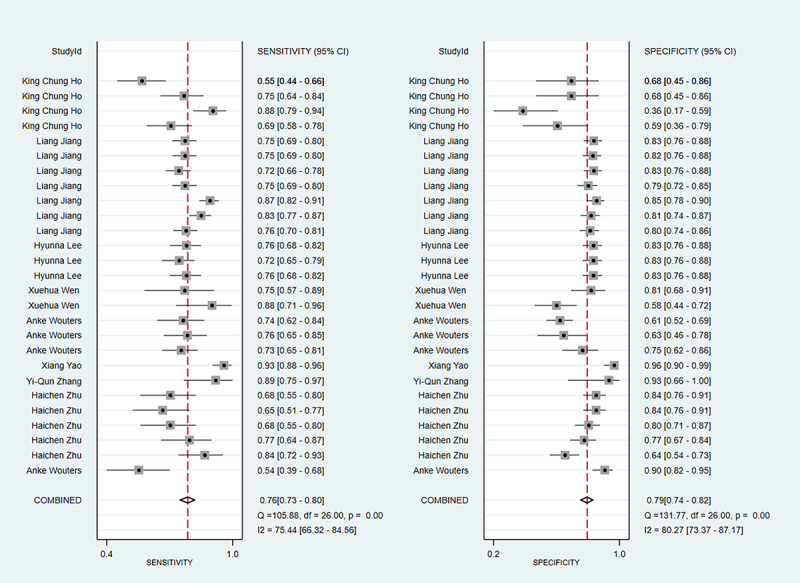
The sensitivity and specificity of the training set for the value of applying machine learning in predicting the time of symptom onset in stroke patients. Some studies used multiple different types of machine learning models; therefore, some studies are presented multiple times in the figure [17, 18, 24-34].

**Figure 4:**
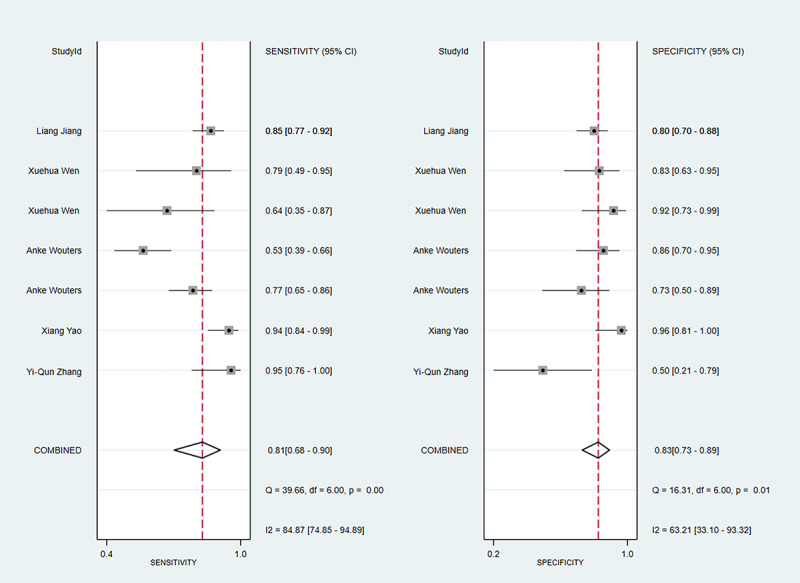
The sensitivity and specificity of the validation set for the value of applying machine learning in predicting the time of symptom onset in stroke patients. Some studies used multiple different types of machine learning models; therefore, some studies are presented multiple times in the figure [17, 18, 24-34].

The meta-analysis of sensitivity in the training and validation sets included 10 studies and 5 studies, respectively. The sensitivity was 0.76 (95% CI 0.73-0.80) in the training set and 0.81 (95% CI 0.68-0.90) in the validation set. The meta-analysis of specificity in the training and validation sets included 10 and 5 studies, respectively. The specificity was 0.79 (95% CI 0.74-0.82) in the training set and 0.83 (95% CI 0.73-0.89) in the validation set. The sensitivity and specificity in the training set are presented in Figure 3; the sensitivity and specificity in the validation set are shown in Figure 4.

### Subgroup Analysis According to Onset Time

**Figure 5:**
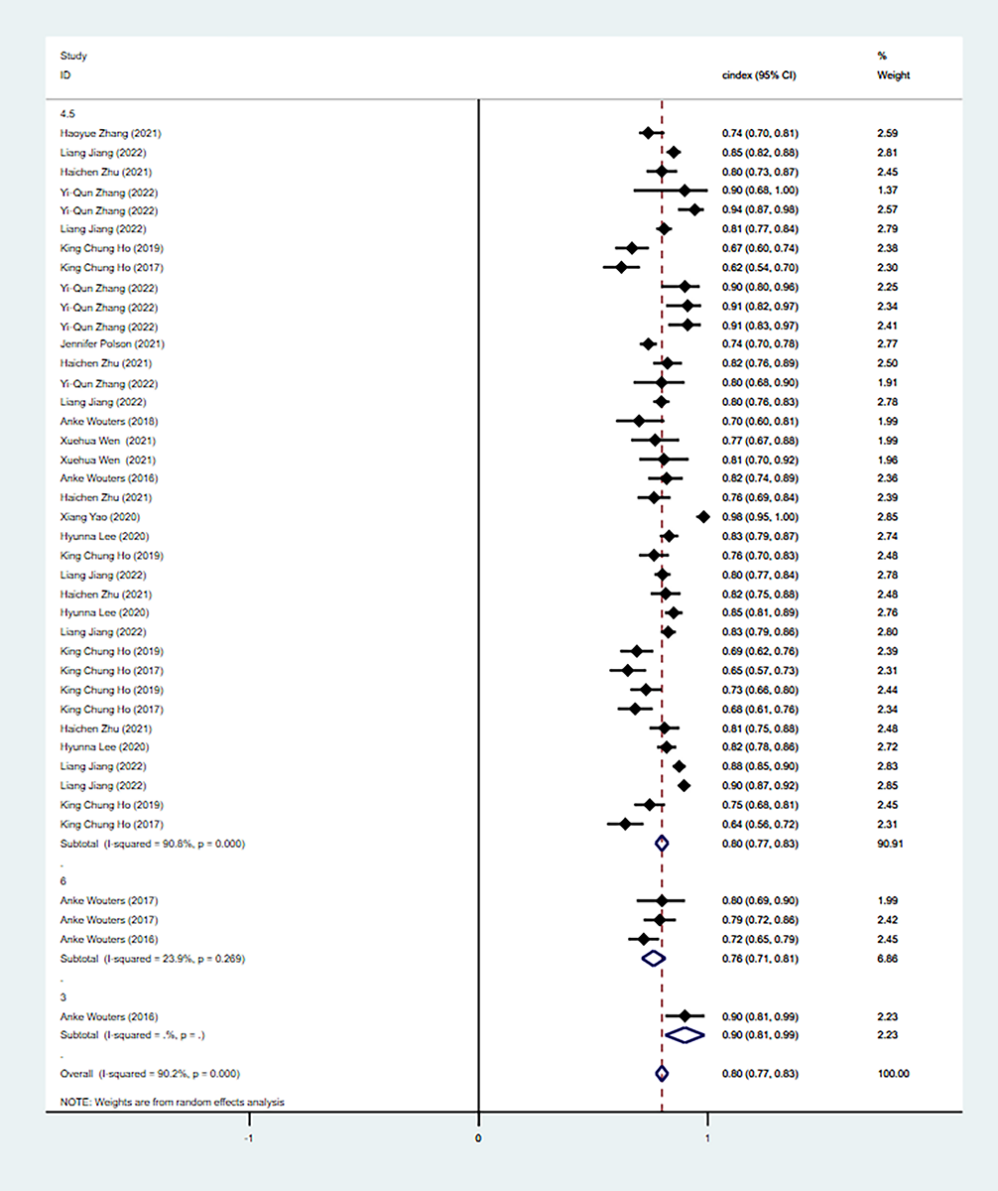
The C index of the training set for the value of applying machine learning in predicting the time of symptom onset in stroke patients. Some studies used multiple different types of machine learning models; therefore, some studies are presented multiple times in the figure [17, 18, 24-34].

**Figure 6:**
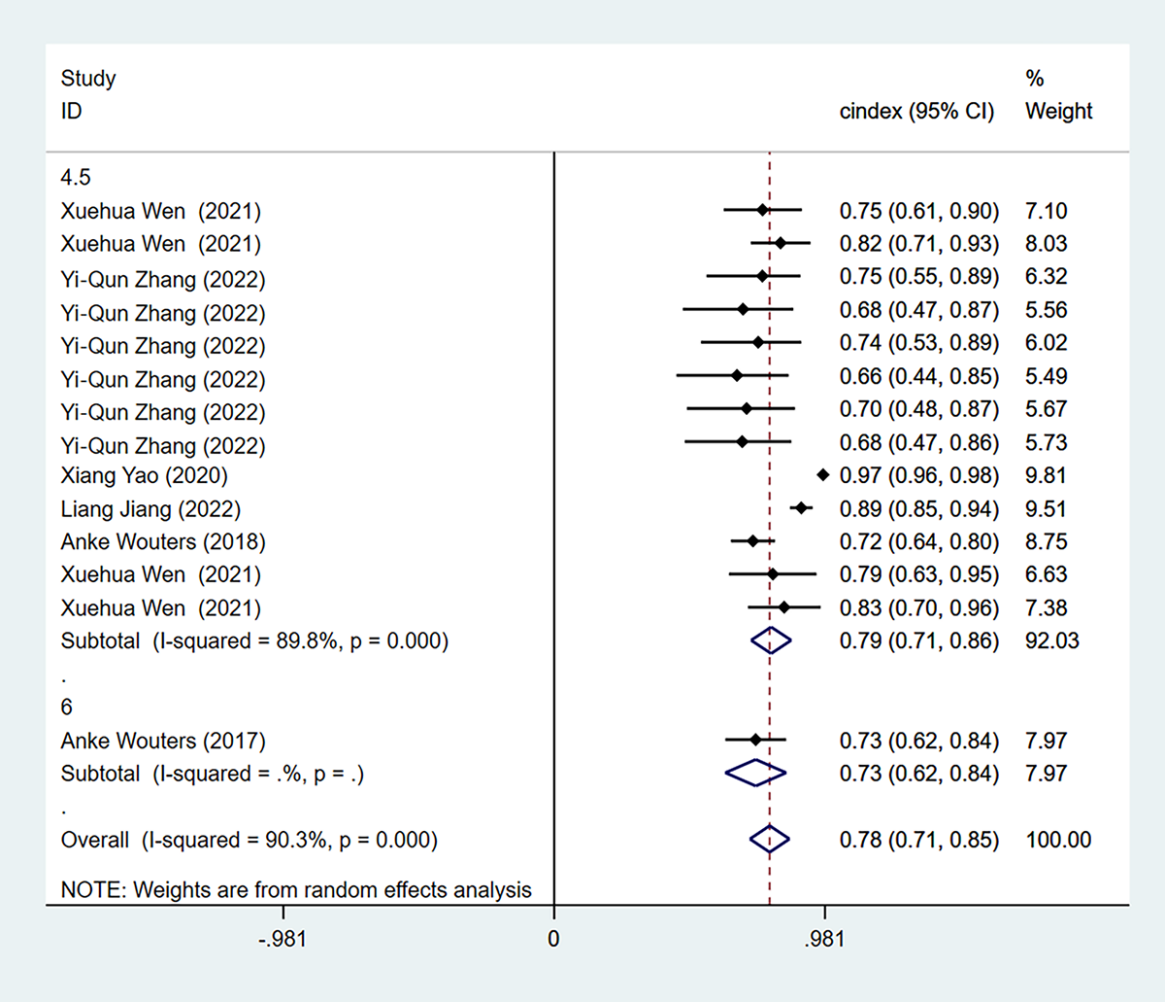
The C index of the verification set for the value of applying machine learning in predicting the time of symptom onset in stroke patients. Some studies used multiple different types of machine learning models; therefore, some studies are presented multiple times in the figure [17, 18, 24-34].

Subgroup analyses of the C index were performed for onset times within 3 hours, 4.5 hours, and 6 hours. The analysis of onset time within 3 hours showed that the C index was 0.90 (95% CI 0.81-0.99) in the training set. For the onset time within 4.5 hours, there were 38 models in the training set and 13 models in the validation set. The C index for 4.5 hours was 0.80 (95% CI 0.77-0.83) in the training set and 0.79 (95% CI 0.71-0.86) in the validation set. Regarding the time within 6 hours, there were 2 models in the training set and 1 in the validation set. The C index for 6 hours was 0.76 (95% CI 0.71-0.81) in the training set and 0.73 (95% CI 0.62-0.84) in the validation set. The C index in the training set is depicted in Figure 5, and the C index of the validation set is presented in Figure 6.

## Discussion

### Principal Findings

Our analysis suggests that machine learning showed ideal performance in predicting and identifying the time of symptom onset in stroke patients. Accurate prediction of stroke onset time is extremely important for identifying the time window of thrombolytic therapy for stroke patients [18]. However, stroke patients usually do not know the specific time of symptom onset. Our research results can provide a reference for the clinical prediction of stroke onset time. We included 10 main types of models, including LR, support vector machine (SVM), artificial neural network (ANN), boosting, deep learning (DL), random forest (RF), decision tree (DT), generalized linear model (GLM), k-nearest neighbor (KNN), and naive Bayes (NB). Generally, the models showed ideal predictive performance, with an overall C index of 0.800 (95% CI 0.773-0.826) in the training set and 0.781 (95% CI 0.709-0.852) in the validation set. The overall C index of the LR model for predicting the stroke onset time was relatively high, at 0.807 (95% CI 0.741-0.872) in the training set and 0.807 (95% CI 0.691-0.923) in the validation set, despite high heterogeneity across the studies. These results indicate that the performance of machine learning in identifying stroke onset time is excellent, and it can be used as a potential tool to determine when a stroke begins. Furthermore, the sensitivity and specificity were 0.76 (95% CI 0.73-0.80) and 0.79 (95% CI 0.74-0.82) in the training set, and 0.81 (95% CI 0.68-0.90) and 0.83 (95% CI 0.73-0.89) in the validation set.

Machine learning models have been widely used in the health care industry and show favorable performance in the stratification of patients. Numerous studies have been carried out on the use of machine learning models to predict the risk of stroke, the prognosis of acute stroke, and motor function recovery in stroke patients after treatment, as well as for the early identification of ischemic stroke patients at a high risk of recurrence [21,35-37]. Despite many publications on outcomes and treatment response in stroke patients, the use of MRI to determine the time of symptom onset is rarely researched. Therefore, this study explored the value of applying machine learning in predicting stroke onset time.

In our study, the variable included in the model for predicting the time of stroke onset was MRI, specifically diffusion-weighted imaging (DWI) and fluid-attenuated inversion recovery (FLAIR). A mismatch between DWI and FLAIR has been adopted to identify stroke patients who are likely to benefit from thrombolysis therapy [38,39]. Previous studies have reported that DWI-FLAIR mismatch could achieve a specificity of 0.60 to 0.80 and a moderate sensitivity of 0.5 to 0.6 [40-42]. Machine learning is a branch of artificial intelligence that can use a variety of image features, including those invisible to humans, with a favorable predictive accuracy [16,18,43].

In addition, our research shows that the LR model had the best predictive performance among the included models. Moreover, some institutions that specialize in detecting tumors have constructed LR-based machine learning models, which have achieved good results [44-46]. Based on existing research and our results, we vigorously recommend promoting the development of nomograms based on LR models for research on multiple diseases.

This study is the first meta-analysis to investigate the value of applying machine learning in predicting the time of symptom onset in stroke patients, comprehensively promoting the development of accurate treatment of stroke. At the same time, it will be useful for identifying stroke onset time once its feasibility is confirmed. However, there are still some limitations to our review. First, the included studies are mainly single-center, and it is difficult to eliminate the impact of socioeconomic background. Second, although we have conducted a comprehensive search, the number of included studies is still small. Third, the diversity of the included models resulted in heterogeneity. Since it is difficult to avoid heterogeneity in machine learning models, more large-scale, multicenter studies are desired to reduce heterogeneity.

### Conclusion

Machine learning has ideal performance in identifying stroke onset time, and it can be used as a potential tool to determine when a stroke begins. Nonetheless, its predictive accuracy still needs to be improved. As a result, minimally invasive or noninvasive, easily collected, and efficient predictors should be considered in future research to improve the predictive accuracy of machine learning. Moreover, it is necessary to explore reasonable image-segmentation and texture-extraction methods in terms of radiomics. In addition, comprehensive research on the popularization of machine learning and the value of its application is desired. Furthermore, the promotion and application of machine learning should take into account differences in ethnic backgrounds.

## Appendix

Multimedia Appendix 1 Literature search strategy.

Multimedia Appendix 2 PRISMA (Preferred Reporting Items for Systematic reviews and Meta-Analyses) checklist.

## Abbreviations

ANN: artificial neural network
CT: computed tomography
DL: deep learning
DT: decision tree
DWI: fluid-attenuated inversion recovery
FLAIR: diffusion-weighted imaging
GLM: generalized linear model
KNN: k-nearest neighbor
LR: logistic regression
MRI: magnetic resonance imaging
NB: naive Bayes
PRISMA: Preferred Reporting Items for Systematic reviews and Meta-Analyses
PROBAST: Prediction Model Risk of Bias Assessment Tool
RF: random forest
SVM: support vector machine

## Data Availability

The data sets generated during and/or analyzed during this study are available from the corresponding author on reasonable request.
