# Supplementary material for: The Value of Applying Machine Learning in Predicting the Time of Symptom Onset in Stroke Patients: Systematic Review and Meta-Analysis
Source: J Med Internet Res. 2023 Oct 12;25:e44895. doi: 10.2196/44895 (PMC10603565; doi:10.2196/44895)
Supplement: Multimedia Appendix 1 [file jmir_v25i1e44895_app1.docx]

Table S1 Literature search strategy

**1.Pubmed**

| Search number | Query | Results |
| --- | --- | --- |
| #1 | "Stroke"[Mesh] | 161276 |
| #2 | (((((((((((((((((((((((((((((((Stroke[Title/Abstract]) OR (Strokes[Title/Abstract])) OR (Cerebrovascular Accident[Title/Abstract])) OR (Cerebrovascular Accidents[Title/Abstract])) OR (Vascular Accident, Brain[Title/Abstract])) OR (Brain Vascular Accident[Title/Abstract])) OR (Brain Vascular Accidents[Title/Abstract])) OR (Vascular Accidents, Brain[Title/Abstract])) OR (Apoplexy[Title/Abstract])) OR (accident, cerebrovascular[Title/Abstract])) OR (acute cerebrovascular lesion[Title/Abstract])) OR (acute focal cerebral vasculopathy[Title/Abstract])) OR (apoplexia[Title/Abstract])) OR (blood flow disturbance, brain[Title/Abstract])) OR (brain accident[Title/Abstract])) OR (brain attack[Title/Abstract])) OR (brain blood flow disturbance[Title/Abstract])) OR (brain insult[Title/Abstract])) OR (brain insultus[Title/Abstract])) OR (cerebral insult[Title/Abstract])) OR (cerebral vascular insufficiency[Title/Abstract])) OR (cerebro vascular accident[Title/Abstract])) OR (cerebrovascular arrest[Title/Abstract])) OR (cerebrovascular failure[Title/Abstract])) OR (cerebrovascular injury[Title/Abstract])) OR (cerebrovascular insufficiency[Title/Abstract])) OR (cerebrovascular insult[Title/Abstract])) OR (cerebrum vascular accident[Title/Abstract])) OR (cryptogenic stroke[Title/Abstract])) OR (CVA[Title/Abstract])) OR (insultus cerebralis[Title/Abstract])) OR (ischaemic seizure[Title/Abstract]) | 316671 |
| #3 | ("Stroke"[Mesh]) OR ((((((((((((((((((((((((((((((((Stroke[Title/Abstract]) OR (Strokes[Title/Abstract])) OR (Cerebrovascular Accident[Title/Abstract])) OR (Cerebrovascular | 352937 |
| #4 | "Machine Learning"[Mesh] | 46744 |
| #5 | ((((((((((((((((machine learning[Title/Abstract]) OR (Transfer Learning[Title/Abstract])) OR (Deep learning[Title/Abstract])) OR (Prediction model[Title/Abstract])) OR (artificial | 809208 |
| #6 | ("Machine Learning"[Mesh]) OR (((((((((((((((((machine learning[Title/Abstract]) OR (Transfer Learning[Title/Abstract])) OR (Deep learning[Title/Abstract])) OR (Prediction | 814686 |
| #7 | (("Stroke"[Mesh]) OR ((((((((((((((((((((((((((((((((Stroke[Title/Abstract]) OR (Strokes[Title/Abstract])) OR (Cerebrovascular Accident[Title/Abstract])) OR (Cerebrovascular Accidents[Title/Abstract])) OR (Vascular Accident, Brain[Title/Abstract])) OR (Brain Vascular Accident[Title/Abstract])) OR (Brain Vascular Accidents[Title/Abstract])) OR (Vascular Accidents, Brain[Title/Abstract])) OR (Apoplexy[Title/Abstract])) OR (accident, cerebrovascular[Title/Abstract])) OR (acute cerebrovascular lesion[Title/Abstract])) OR (acute focal cerebral vasculopathy[Title/Abstract])) OR (apoplexia[Title/Abstract])) OR (blood flow disturbance, brain[Title/Abstract])) OR (brain accident[Title/Abstract])) OR (brain attack[Title/Abstract])) OR (brain blood flow disturbance[Title/Abstract])) OR (brain insult[Title/Abstract])) OR (brain insultus[Title/Abstract])) OR (cerebral insult[Title/Abstract])) OR (cerebral vascular insufficiency[Title/Abstract])) OR (cerebro vascular accident[Title/Abstract])) OR (cerebrovascular arrest[Title/Abstract])) OR (cerebrovascular failure[Title/Abstract])) OR (cerebrovascular injury[Title/Abstract])) OR (cerebrovascular insufficiency[Title/Abstract])) OR (cerebrovascular insult[Title/Abstract])) OR (cerebrum vascular accident[Title/Abstract])) OR (cryptogenic stroke[Title/Abstract])) OR (CVA[Title/Abstract])) OR (insultus cerebralis[Title/Abstract])) OR (ischaemic seizure[Title/Abstract]))) AND (("Machine Learning"[Mesh]) OR (((((((((((((((((machine learning[Title/Abstract]) OR (Transfer Learning[Title/Abstract])) OR (Deep learning[Title/Abstract])) OR (Prediction model[Title/Abstract])) OR (artificial intelligence[Title/Abstract])) OR (random forest[Title/Abstract])) OR (artificial neural network[Title/Abstract])) OR (ANN[Title/Abstract])) OR (Support vector machine[Title/Abstract])) OR (SVM[Title/Abstract])) OR (Gradient Boosting Machine[Title/Abstract])) OR (GBM[Title/Abstract])) OR (Nomogram[Title/Abstract])) OR (XGboost[Title/Abstract])) OR (Logistic[Title/Abstract])) OR (Decision tree[Title/Abstract])) OR (validation[Title/Abstract]))) | 21887 |
|  | ((onset time[Title/Abstract]) OR (onset[Title/Abstract])) OR (Stroke time[Title/Abstract]) | 558695 |
|  | ((("Stroke"[Mesh]) OR ((((((((((((((((((((((((((((((((Stroke[Title/Abstract]) OR (Strokes[Title/Abstract])) OR (Cerebrovascular Accident[Title/Abstract])) OR (Cerebrovascular Accidents[Title/Abstract])) OR (Vascular Accident, Brain[Title/Abstract])) OR (Brain Vascular Accident[Title/Abstract])) OR (Brain Vascular Accidents[Title/Abstract])) OR (Vascular Accidents, Brain[Title/Abstract])) OR (Apoplexy[Title/Abstract])) OR (accident, cerebrovascular[Title/Abstract])) OR (acute cerebrovascular lesion[Title/Abstract])) OR (acute focal cerebral vasculopathy[Title/Abstract])) OR (apoplexia[Title/Abstract])) OR (blood flow disturbance, brain[Title/Abstract])) OR (brain accident[Title/Abstract])) OR (brain attack[Title/Abstract])) OR (brain blood flow disturbance[Title/Abstract])) OR (brain insult[Title/Abstract])) OR (brain insultus[Title/Abstract])) OR (cerebral insult[Title/Abstract])) OR (cerebral vascular insufficiency[Title/Abstract])) OR (cerebro vascular accident[Title/Abstract])) OR (cerebrovascular arrest[Title/Abstract])) OR (cerebrovascular failure[Title/Abstract])) OR (cerebrovascular injury[Title/Abstract])) OR (cerebrovascular insufficiency[Title/Abstract])) OR (cerebrovascular insult[Title/Abstract])) OR (cerebrum vascular accident[Title/Abstract])) OR (cryptogenic stroke[Title/Abstract])) OR (CVA[Title/Abstract])) OR (insultus cerebralis[Title/Abstract])) OR (ischaemic seizure[Title/Abstract]))) AND (("Machine Learning"[Mesh]) OR (((((((((((((((((machine learning[Title/Abstract]) OR (Transfer Learning[Title/Abstract])) OR (Deep learning[Title/Abstract])) OR (Prediction model[Title/Abstract])) OR (artificial intelligence[Title/Abstract])) OR (random forest[Title/Abstract])) OR (artificial neural network[Title/Abstract])) OR (ANN[Title/Abstract])) OR (Support vector machine[Title/Abstract])) OR (SVM[Title/Abstract])) OR (Gradient Boosting Machine[Title/Abstract])) OR (GBM[Title/Abstract])) OR (Nomogram[Title/Abstract])) OR (XGboost[Title/Abstract])) OR (Logistic[Title/Abstract])) OR (Decision tree[Title/Abstract])) OR (validation[Title/Abstract])))) AND (((onset time[Title/Abstract]) OR (onset[Title/Abstract])) OR (Stroke time[Title/Abstract])) | 2778 |

**2.Cochrane**

| Search number | Query | Results |
| --- | --- | --- |
| #1 |  |  |
| #2 |  |  |
| #3 |  |  |
| #4 |  |  |
| #5 |  |  |
| #6 |  |  |
| #7 |  |  |
| #8 |  |  |
| #9 |  |  |
| #10 |  |  |
| #11 |  |  |
| #12 |  |  |
| #13 |  |  |
| #14 |  |  |
| #15 |  |  |
| #16 |  |  |
| #17 |  |  |
| #18 |  |  |
| #19 |  |  |
| #20 |  |  |
| #21 |  |  |
| #22 |  |  |

**3.Embase**

| Search number | Query | Results |
| --- | --- | --- |
| #1 |  |  |
| #2 |  |  |
| #3 |  |  |
| #4 |  |  |
| #5 |  |  |
| #6 |  |  |
| #7 |  |  |
| #8 |  |  |
| #9 |  |  |
| #10 |  |  |

**4.Web of science**

| Search number | Query | Results |
| --- | --- | --- |
| #1 | Stroke (主题) or Strokes (主题) or Cerebrovascular Accident (主题) or Cerebrovascular Accidents (主题) or Vascular Accident, Brain (主题) or Brain Vascular Accident (主题) or Brain Vascular Accidents (主题) or Vascular Accidents, Brain (主题) or Apoplexy (主题) or accident, cerebrovascular (主题) or acute cerebrovascular lesion (主题) or acute focal cerebral vasculopathy (所有字段) or apoplexia (主题) or blood flow disturbance, brain (主题) or brain accident (主题) or brain blood flow disturbance (主题) or brain insult (主题) or brain insultus (主题) or cerebral insult (主题) or cerebral vascular insufficiency (主题) or cerebro vascular accident (主题) or cerebrovascular arrest (主题) or cerebrovascular failure (主题) or cerebrovascular injury (主题) or cerebrovascular insufficiency (主题) or cerebrovascular insult (主题) or cerebrum vascular accident (主题) or cryptogenic stroke (主题) or CVA (主题) or insultus cerebralis (主题) or ischaemic seizure (主题) or brain attack (主题) | 454572 |
| #2 | machine learning (主题) or Transfer Learning (主题) or Deep learning (主题) or Prediction model (主题) or artificial intelligence (主题) or random forest (主题) or artificial neural network (主题) or ANN (主题) or Support vector machine (主题) or SVM (主题) or Gradient Boosting Machine (主题) or GBM (所有字段) or Nomogram (主题) or XGboost (主题) or Logistic (主题) or Decision tree (主题) or validation (主题) | 2656670 |
| #3 | onset time (主题) or onset (主题) or Stroke time (主题) | 747749 |
| #4 | #1 AND #2 AND #3 | 10797 |
